# Higher-Protein Nutrition and Concurrent Exercise in Obesity: A Narrative Review of Body Composition, Metabolic Health, and Physical Function

**DOI:** 10.3390/nu18142280

**Published:** 2026-07-11

**Authors:** Claudia Reytor-González, Andrés Loor-Cedeño, Dolores Jima Gavilanes, Andri Matos, Juan Marcos Parise-Vasco, Jaime Angamarca-Iguago, Jaen Cagua-Ordoñez, Martín Campuzano-Donoso, Daniel Simancas-Racines

**Affiliations:** 1 Center for Evidence Ecosystems, Implementation Science, and Decision-Making (CIDES), Facultad de Ciencias de la Salud y Bienestar Humano, Universidad Tecnológica Indoamérica, Ambato 180150, Ecuador; 2 Independent Researcher, Santo Domingo 230101, Ecuador; 3 Escuela de Medicina, Universidad Espíritu Santo, Samborondón 0901952, Ecuador; 4 School of Allied Health, Eastwick College, Ramsey, NJ 07446, USA; 5 Center for Evidence Ecosystems, Implementation Science, and Decision-Making (CIDES), Facultad de Ciencias de la Salud y Bienestar Humano, Universidad Tecnológica Indoamérica, Quito 170103, Ecuador

**Keywords:** obesity, higher-protein nutrition, concurrent exercise, resistance training, aerobic training, sarcopenic obesity, body composition, metabolic health, physical function, narrative review

## Abstract

Obesity care increasingly requires outcomes that extend beyond total body weight. Although weight reduction remains clinically meaningful, the scale alone does not indicate whether fat mass, lean tissue, muscle quality, strength, mobility, or physiological reserve have changed in a favorable direction. This narrative review examines the combined role of higher-protein dietary strategies and concurrent exercise in adults with obesity, with emphasis on body composition, metabolic health, and physical function. Higher-protein dietary strategies may support satiety, improve the tolerability of energy restriction in some patients, and attenuate lean-tissue loss during weight reduction, while evidence for superior long-term weight loss remains inconsistent. Exercise provides complementary stimuli to protein-focused nutrition: resistance training supports strength, muscle function, and lean-tissue preservation, while aerobic and interval-based training contribute to cardiorespiratory fitness, regional adiposity reduction, and selected cardiometabolic adaptations. Concurrent training offers a practical framework for integrating these stimuli, with value primarily as multidomain coverage rather than as evidence of universal superiority over other modalities. The main rationale for combining protein adequacy with exercise is preservation of usable physical function during weight loss, particularly in older adults, patients with sarcopenic-obesity risk, and individuals undergoing rapid pharmacological or surgical weight reduction. Overall, the available evidence is most consistent with viewing higher-protein nutrition and structured exercise as complementary strategies for improving the quality of weight loss, rather than as a single protocol for maximizing scale-weight reduction. Longer pragmatic trials are needed to clarify phenotype-specific responses, feasible protein targets, exercise progression, monitoring strategies, and functional outcomes.

## 1. Introduction

Obesity is increasingly understood as a chronic disease of excess or dysfunctional adiposity, rather than as a simple increase in body weight [1,2]. Body-mass index (BMI), although useful for screening and population surveillance, does not determine how adipose tissue is distributed, whether ectopic lipid has accumulated, or whether excess adiposity is already associated with cardiometabolic impairment, reduced physical function, or loss of physiological reserve in an individual patient [1]. Contemporary clinical-obesity frameworks therefore place greater emphasis on adiposity-related biological and functional impairment than on the crossing of a single anthropometric threshold [1]. This clinical framework shifts the endpoint of obesity care: weight loss remains clinically meaningful, but the scale alone does not show which tissue compartments have changed or whether the intervention has improved the patient’s functional capacity [3,4]. However, this broader framing also raises an evidentiary challenge: many obesity interventions still report weight-centric outcomes more consistently than tissue-specific, performance-based, or patient-centered functional outcomes, limiting how confidently clinicians can judge the quality of weight loss [3,4].

This shift is especially important in older adults, patients with sarcopenic-obesity risk, and individuals who begin treatment with low strength, limited mobility, or reduced exercise tolerance [3,4,5,6]. In these groups, the composition and consequences of weight loss can be as clinically relevant as its magnitude. A similar reduction in body weight may represent preferential fat-mass loss, disproportionate lean-tissue loss, preserved strength, improved mobility, or no measurable functional gain [3,4]. The same concern has become more visible with the use of highly effective anti-obesity pharmacotherapies, including glucagon-like peptide-1 (GLP-1) receptor agonists and dual glucose-dependent insulinotropic polypeptide (GIP)/(GLP-1) receptor agonists [7,8,9]. These agents can produce substantial reductions in body weight and fat mass, while body-composition analyses indicate that lean mass may also decline during high-magnitude weight loss [7,8,9]. These findings require interpretation in relation to baseline phenotype, age, body-composition method, muscle quality, strength, and physical function, because these factors may modify the clinical meaning of lean-mass decline [4,7,8,9]. At present, the clinical significance of lean-mass reduction during pharmacological or surgical weight loss remains insufficiently resolved because most studies do not simultaneously characterize muscle quality, strength, physical performance, dietary intake, and training exposure with enough granularity [4,7,8,9].

Skeletal muscle is central to that interpretation because it contributes to locomotor capacity, postprandial glucose disposal, physical independence, and resilience during illness or aging [4,5,6]. Sarcopenic obesity illustrates the limitation of weight-centered thinking particularly well: the phenotype combines excess adiposity with low skeletal-muscle mass and impaired muscle function, making function part of the diagnostic construct rather than an optional secondary outcome [5,6]. For this reason, preserving lean tissue is relevant but insufficient. Measured lean mass does not necessarily represent contractile muscle quality, strength, mobility, or physical performance; obesity interventions are therefore better evaluated using both tissue compartments and indicators of movement, exertional tolerance, and function after weight reduction [3,4,5,6]. The distinction determines whether an intervention is interpreted as metabolically successful, functionally protective, or potentially incomplete despite producing clinically meaningful weight loss.

Exercise and protein-focused nutrition address this problem through complementary pathways. Structured exercise can improve cardiorespiratory fitness, body composition, physical function, and selected cardiometabolic outcomes in adults with overweight or obesity, even when changes in scale weight are modest [10,11,12,13]. Aerobic training is especially relevant for cardiorespiratory fitness and adiposity-related outcomes, whereas resistance training provides the mechanical stimulus needed for strength, muscle function, and lean-tissue preservation during weight management [12,13,14,15,16]. Concurrent training brings these stimuli into the same intervention framework; its value in obesity care lies in addressing multiple treatment domains within one program [12,13,17,18,19]. Protein adequacy provides the nutritional counterpart to this training stimulus. Higher-protein dietary strategies may help attenuate lean-tissue loss during energy restriction and may support adaptation to resistance exercise [20,21]. They may also improve satiety or make energy restriction more tolerable for some patients, although their long-term effects on total weight loss are variable [21,22]. A more evidence-concordant interpretation is that protein adequacy may improve the composition and functional interpretation of weight loss when integrated with progressive resistance or concurrent training, rather than making higher-protein diets universally superior weight-loss diets [20,21]. This interpretation remains cautious because mechanistic plausibility and short-term body-composition changes do not by themselves establish durable functional benefit, and available trials differ substantially in protein dose, achieved intake, exercise supervision, adherence, and outcome selection [21,23,24].

The evidence base for this combined approach remains heterogeneous. Trials differ in protein target, achieved intake, energy deficit, exercise modality, supervision, adherence, outcome selection, and patient phenotype [21,23,24]. Some emphasize body weight, whereas others focus on lean-mass retention, fitness, metabolic markers, or physical function [21,23,24]. This heterogeneity limits direct comparison across interventions and makes it inappropriate to infer a single optimal protein-exercise prescription for all adults with obesity. A narrative synthesis can accommodate this heterogeneity because a single optimal protocol is not inferable from such varied evidence. The review therefore clarifies how protein intake and concurrent exercise may interact with body composition, metabolic health, and functional outcomes, while distinguishing direct, indirect, phenotype-specific, and uncertain evidence. This review therefore examines the combined role of higher-protein dietary strategies and concurrent exercise in adults with obesity, with the aim of synthesizing the clinical and physiological rationale for pairing these approaches, identifying practical implications, and defining research priorities for interventions designed to improve the quality—not only the quantity—of weight loss.

## 2. Methods

This manuscript was developed as a narrative review with a clinical and physiological focus. The review aimed to synthesize evidence on higher-protein dietary strategies and concurrent exercise in adults with overweight or obesity, with emphasis on body composition, metabolic health, physical function, and clinical implementation rather than to provide quantitative effect estimates or graded recommendations.

A targeted literature search was conducted on 20 May 2026 in PubMed/MEDLINE, Scopus, and the Cochrane Library. Additional searches were performed in Google Scholar and ResearchGate to identify relevant grey literature, preprints, clinical documents, and articles not retrieved through database searches. The reference lists of included articles and key reviews were also screened manually to identify additional sources of conceptual, clinical, or methodological relevance.

Search terms included combinations of: “obesity”, “overweight”, “high-protein diet”, “protein intake”, “protein adequacy”, “weight loss”, “energy restriction”, “resistance training”, “aerobic exercise”, “high-intensity interval training”, “concurrent training”, “body composition”, “lean mass”, “fat mass”, “skeletal muscle”, “muscle strength”, “physical function”, “sarcopenic obesity”, “metabolic health”, “GLP-1 receptor agonists”, “tirzepatide”, “bariatric surgery”, and “chronic kidney disease”. Boolean operators were used to combine nutrition-, exercise-, obesity-, and outcome-related terms.

Potentially relevant sources were reviewed by the authors for conceptual, clinical, and methodological relevance to the scope of the manuscript. Priority was given to clinical guidelines, consensus statements, systematic reviews, meta-analyses, randomized controlled trials, and methodological papers addressing protein intake, exercise training, body composition or functional outcomes in adults with overweight or obesity. Publications from the previous five years were prioritized, although older studies were retained when they represented seminal evidence, foundational physiology, widely cited guidance, or relevant methodological work.

Sources were considered eligible when they addressed at least one of the main domains of the review: protein intake or distribution during weight loss; resistance, aerobic, interval, or concurrent exercise; fat mass, lean mass, skeletal-muscle mass, muscle quality, regional adiposity, cardiometabolic markers, physical function, adherence, tolerability, or clinical implementation. Evidence from adjacent populations, including older adults, individuals with sarcopenic obesity, patients receiving incretin-based pharmacotherapy, bariatric-surgery populations, patients with chronic kidney disease, and individuals with type 2 diabetes or metabolic dysfunction-associated steatotic liver disease, was included when it helped interpret obesity-related mechanisms, clinical risks, or implementation issues. Such evidence was interpreted as supportive rather than as direct evidence for all adults with obesity.

The synthesis was narrative and interpretive rather than statistical. Evidence was organized around the main clinical questions of the review: the limitations of body weight as a treatment endpoint; the role of higher-protein diets during weight loss; the effects of different exercise modalities on body composition, metabolic health, and physical function; and the rationale for combining protein adequacy with concurrent exercise. Because this was not designed as a systematic review or meta-analysis, no protocol was registered, PRISMA reporting was not applied, no duplicate screening or duplicate data extraction was performed, no formal risk-of-bias assessment was executed, and findings were not quantitatively pooled or graded. Conclusions were therefore framed as clinically oriented interpretations of the available evidence rather than as definitive comparative effectiveness estimates or graded recommendations.

## 3. Obesity, Body Composition, and Functional Decline

Obesity is still often interpreted through body weight, although its clinical burden is better explained by the interaction between excess adiposity, altered tissue function, body-composition change, and declining physiological reserve [1,3,4]. BMI remains useful for screening, but it cannot distinguish visceral from subcutaneous fat, quantify ectopic fat, or determine whether a patient has poor muscle quality, low relative strength, reduced exercise tolerance, or impaired mobility [1,3]. This limitation is clinically relevant because contemporary definitions of clinical obesity emphasize adiposity-related biological or functional impairment rather than excess body mass alone [1]. When obesity is approached as a disease of altered tissue function, evaluation of treatment success includes both compartmental change and whether the patient becomes metabolically and functionally more resilient [3,4]. However, this broader interpretation also requires more demanding outcome assessment. A reduction in weight or BMI may be clinically useful, but it is an incomplete surrogate when the intervention is intended to improve adiposity-related risk, preserve muscle-related function, or protect physiological reserve.

Excess adiposity affects more than adipose tissue. It can modify skeletal-muscle performance, movement efficiency, cardiometabolic regulation, and the energetic cost of daily activity [4,5,6]. Many adults with obesity have normal or elevated absolute lean mass, yet lower strength relative to body size, poorer muscle quality, greater joint loading, and higher locomotor demand [4,5,6]. Insulin resistance, chronic low-grade inflammation, myosteatosis, reduced mitochondrial efficiency, pain, dyspnea, and physical inactivity may further impair performance in some phenotypes [4,5,6,10,11,25,26]. A patient may therefore appear to have adequate lean mass on densitometry while still showing limited walking tolerance, early fatigue, slow chair-rise performance, poor gait speed, or low functional reserve [3,4,5,6]. Assessment therefore extends from measurement of lean tissue to whether the measured tissue corresponds to contractile quality, relative strength, mobility, and usable physical capacity.

Sarcopenic obesity provides a clear example. The consensus statement from the European Society for Clinical Nutrition and Metabolism (ESPEN) and the European Association for the Study of Obesity (EASO) defines sarcopenic obesity by the coexistence of excess adiposity, low skeletal-muscle mass, and impaired muscle function, thereby placing function within the phenotype rather than treating it as an optional outcome [5,6]. This framework helps identify at-risk patients who may be missed when assessment relies mainly on BMI, total body weight, or crude estimates of lean mass [5,6]. Prevalence estimates vary according to diagnostic criteria and measurement methods, but the construct remains clinically important because it identifies a subgroup in whom weight reduction without attention to muscle and function may be incomplete, poorly tolerated, or potentially harmful [5,27]. At the same time, the field still lacks full diagnostic uniformity, and this limits comparison across studies. Sarcopenic-obesity research is therefore interpreted with attention to the operational definition used, the method used to estimate muscle mass or adiposity, and whether functional impairment was actually measured rather than assumed.

**Figure 1:**
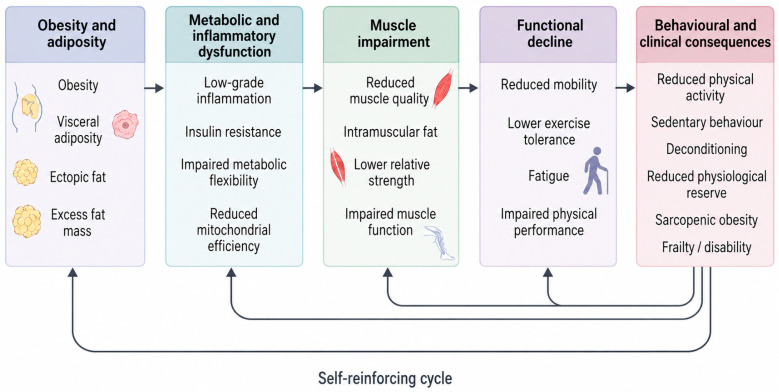
Conceptual pathway from obesity to functional decline. Excess visceral and ectopic adiposity may contribute to insulin resistance, low-grade inflammation, reduced skeletal-muscle efficiency, and impaired physical tolerance; these interacting factors may be associated with lower muscle quality, poorer relative strength, reduced mobility, lower exercise tolerance, and further sedentary behavior [1,5,6,10,11,25,26,28,29,30,31]. The figure is presented as a conceptual model of interacting pathways rather than as a causal sequence established uniformly across all obesity phenotypes. The self-reinforcing cycle is intended to indicate that behavioral and clinical consequences may feed back not only to adiposity, but also to metabolic dysfunction, muscle impairment, and functional decline.

Functional decline in obesity can also develop before advanced age or overt disability. Excess adiposity, pain, breathlessness, low fitness, movement avoidance, sedentary behavior, and deconditioning can reinforce one another over time [10,11,25,26]. As movement becomes more effortful, daily activity may decrease; as activity decreases, strength, fitness, confidence, and mobility may decline further [10,11]. This pathway supports viewing physical activity recommendations in obesity as more than strategies to increase energy expenditure. They also serve to preserve mobility, confidence, cardiometabolic health, and independence, particularly when weight loss occurs in patients who already have low reserve [10,11,12,13]. Figure 1 summarizes this conceptual pathway linking excess adiposity, metabolic dysfunction, skeletal-muscle impairment, and progressive functional decline. Nevertheless, the direction and magnitude of these relationships are not uniform across all patients. Some evidence is observational or phenotype-dependent, and functional limitation may be driven by comorbid pain, osteoarthritis, cardiopulmonary disease, mental health, environmental barriers, or weight stigma as much as by adiposity itself.

The same perspective exposes a persistent weakness in weight-centered obesity care. Similar reductions in body weight may reflect very different combinations of fat-mass loss, lean-tissue loss, fluid change, regional adiposity reduction, strength preservation, or functional improvement [3,4]. An intervention that reduces visceral or ectopic fat while preserving strength and mobility has a different clinical meaning from one that produces comparable scale-weight reduction with greater lean-tissue loss or no improvement in physical capacity [3,4]. This issue has become more visible in the era of highly effective anti-obesity pharmacotherapy. GLP-1 receptor agonists and dual incretin therapies can produce substantial weight loss, while body-composition analyses show that lean mass may also decline during treatment [7,8,9]. Some lean-tissue reduction is expected when body size decreases substantially, but its clinical meaning depends on baseline phenotype, age, magnitude and rate of weight loss, body-composition method, muscle quality, strength, and physical function [4,7,8,9]. The current evidence neither establishes that pharmacological weight loss causes clinically harmful muscle wasting nor that lean-mass loss is functionally irrelevant. A central unresolved issue is whether tissue loss is proportional, modifiable, and accompanied by preserved or improved function.

The SURMOUNT-1 body-composition substudy illustrates the need for that interpretation. Tirzepatide produced large reductions in total body weight and fat mass over 72 weeks, while lean mass also declined [9]. These findings, in isolation, do not support either alarmist or reassuring interpretations. They suggest that modern pharmacological treatment can produce favorable fat-mass reduction, but they also reinforce the need to monitor tissue partitioning and functional outcomes during high-magnitude weight loss [7,8,9]. Clinically, interpretation depends on whether lean-mass change is disproportionate, functionally meaningful, or potentially modifiable through nutrition, resistance training, aerobic conditioning, and clinical monitoring [12,13].

Exercise is central to this broader standard because many of its benefits are not captured by scale weight. Exercise-induced weight loss may be modest when diet is unchanged, yet structured training can improve cardiorespiratory fitness, strength, physical function, and selected cardiometabolic outcomes in adults with overweight or obesity [13,14,15,16]. Aerobic training is particularly relevant for cardiorespiratory fitness and adiposity-related outcomes, whereas resistance training is central to strength, muscle function, and lean-tissue preservation during weight management [13,14,15,16]. Concurrent training integrates these stimuli and may be useful when treatment goals include fat reduction, metabolic improvement, and functional preservation within the same program, with the role defined as multidomain coverage rather than universal superiority [12,13,17]. The evidence does not support a simplified hierarchy in which one exercise modality is always preferable [17,18,19]. Modality, dose, supervision, adherence, pain, baseline fitness, and clinical phenotype all influence whether exercise changes body composition, cardiometabolic risk, or functional capacity.

Body-composition methodology adds another layer of caution. Dual-energy X-ray absorptiometry (DXA), bioelectrical impedance analysis (BIA), computed tomography (CT), magnetic resonance imaging (MRI), and anthropometric measures do not capture identical constructs, and lean mass is not synonymous with contractile skeletal muscle or muscle quality [3,4]. DXA-derived lean soft tissue includes water, organs, connective tissue, and other non-contractile components, while BIA is sensitive to hydration status and prediction equations [3,4]. Imaging-based methods can provide more detailed information on visceral fat, ectopic fat, skeletal-muscle cross-sectional area, and myosteatosis, but they are not always feasible in routine care [3,4]. For this reason, body-composition measures are interpreted alongside functional outcomes such as grip strength, chair-rise performance, gait speed, stair climbing, cardiorespiratory fitness, fatigue, and patient-reported mobility [4,5,6]. Without these functional measures, apparent preservation of lean mass may overstate clinical benefit, and apparent lean-mass loss may overstate harm. Interpretation depends on the method, the compartment measured, the rate of weight loss, and the patient’s functional trajectory.

Taken together, this evidence supports a treatment model in which the clinical interpretation of weight loss depends on both tissue change and function [1,3,4]. A useful working concept is that higher-quality weight loss preferentially reduces harmful adiposity while preserving skeletal-muscle integrity, cardiometabolic health, and physical capacity [4,12,13]. This model provides the rationale for combining higher-protein nutrition with concurrent exercise: dietary protein may support lean-tissue preservation during energy restriction, while resistance and aerobic training provide mechanical and metabolic stimuli needed to improve muscle function, fitness, and cardiometabolic adaptation [17,21]. This rationale is clinically coherent, but it remains stronger as a framework for integrated care than as proof that a single combined protocol will produce superior outcomes in every obesity phenotype.

## 4. High-Protein Diets in Obesity

### 4.1. Defining High-Protein Diets in Obesity

The term high-protein diet is used inconsistently across obesity trials, clinical nutrition, and sports-nutrition literature [20,21,32]. This creates a practical problem because the same label can describe very different prescriptions depending on whether protein is expressed as grams per day, grams per kilogram of actual body weight, grams per kilogram of adjusted or target body weight, percentage of total energy, or supplement dose [20,21,32]. During hypocaloric treatment, the problem becomes even more apparent: a diet providing 25–30% of energy from protein may correspond to substantially different absolute protein intakes depending on the total energy prescription [21,32]. For this reason, protein targets in obesity care require specification of both the dose and the weight reference used to calculate it [20,21,32].

The adult recommended dietary allowance (RDA) of 0.8 g/kg/day is best interpreted as a minimum intake to prevent deficiency, not as a therapeutic target for preserving lean tissue during energy restriction, rapid weight loss, or progressive resistance training [20,33]. This distinction is important from a public-health perspective because protein intake in many high-income Western populations already meets or exceeds minimum recommendations, whereas excess energy intake, poor overall diet quality, low fiber intake, and suboptimal food patterns remain major nutritional concerns [34,35,36]. Protein needs in obesity are not determined by excess fat mass alone. Actual body weight can overestimate requirements in severe obesity, whereas ideal body weight may underestimate the intake needed during intentional weight loss, especially when baseline intake is low, resistance training is prescribed, or sarcopenic-obesity risk is present [21,32]. In clinical practice, adjusted body weight, target body weight, or estimated fat-free mass may therefore be more useful than actual body weight when available [32]. A practical working range for many adults with obesity undergoing intentional weight loss is approximately 1.2–1.6 g/kg/day using an appropriate weight reference, with higher intakes considered selectively when training status, older age, rapid weight loss, or functional vulnerability increases concern for lean-tissue loss [20,21,32]. This range is not a universal rule; renal status, energy deficit, appetite, gastrointestinal tolerance, food access, dietary pattern, and clinical goals shape the final prescription [32,37]. The prescribed target also differs from the achieved intake, because adherence, appetite suppression, food insecurity, gastrointestinal symptoms, and supplement tolerance may determine the biologically relevant exposure more than the nominal prescription.

### 4.2. Satiety, Energy Balance, and Diet Quality

Higher-protein diets are often justified by their effects on satiety, diet-induced thermogenesis, and energy balance [22]. The physiological rationale is plausible: protein-rich meals can increase fullness and may produce a higher thermic effect than meals dominated by carbohydrate or fat [22]. In obesity care, however, the more important question is whether this translates into better adherence to an energy-restricted dietary pattern over time [21,22]. The evidence is more consistent for appetite support than for a large independent effect on long-term weight loss [21,22]. Some patients may find higher-protein meals useful because they reduce hunger, improve meal structure, or make caloric restriction more tolerable, but high-protein diets are not uniquely effective fat-loss diets [21,22]. Short-term appetite effects, thermogenesis, and favorable macronutrient narratives can be overinterpreted when total energy intake, dietary adherence, and long-term behavioral sustainability are not adequately measured.

Diet quality remains central to this interpretation. A higher-protein pattern that displaces fiber-rich foods, fruits, vegetables, legumes, whole grains, or unsaturated fat sources is not equivalent to one that achieves protein adequacy within a nutrient-dense dietary pattern [20,21]. Protein prescriptions are therefore embedded within the overall diet rather than treated as an isolated macronutrient target [21,38]. When appetite is reduced, food variety narrows, or supplements begin to replace meals, a technically adequate protein intake may still coexist with poor fiber intake, low micronutrient density, or limited cardiometabolic diet quality [20,38]. Consequently, evaluation focuses on whether the total diet remains adequate, cardiometabolically appropriate, culturally acceptable, and sustainable during weight loss, not only on whether protein intake is high.

### 4.3. Lean-Tissue Preservation During Energy Restriction

The main clinical argument for higher protein intake in obesity is attenuation of lean-tissue loss during energy restriction rather than greater scale-weight loss [21]. Weight reduction usually includes some loss of fat-free mass, and the proportion of weight lost as lean tissue becomes more important when weight loss is rapid, the energy deficit is severe, or baseline functional reserve is low [3,4,21]. Enhanced protein intake can attenuate muscle-mass loss during weight-loss interventions in adults with overweight or obesity, although effects on strength and physical function are less consistent [21]. Therefore, evidence showing better lean-mass retention is supportive but incomplete without accompanying measures of strength, physical performance, fatigue, or daily function.

Protein also has limits. It may improve the nutritional environment for lean-tissue preservation during moderate energy restriction, but it is unlikely to fully offset severe caloric restriction, illness, inadequate resistance exercise, low adherence, or rapid treatment-induced weight loss [12,13,21,39]. DXA-derived lean soft tissue includes non-contractile components and can shift with hydration and glycogen changes, so body-composition outcomes are interpreted alongside strength, gait speed, chair-rise ability, cardiorespiratory fitness, fatigue, and patient-reported mobility whenever feasible [3,4,5,6]. Clinically, the aim is preservation of usable physical capacity while harmful adiposity is reduced, rather than preservation of a scan-derived lean-mass value in isolation.

### 4.4. Protein Quality, Source, and Distribution

Once total protein intake is adequate, protein quality and distribution may become relevant modifiers, especially in older adults, patients with anabolic resistance, low baseline intake, or sarcopenic-obesity risk [33,40]. High-quality protein sources provide digestible indispensable amino acids, including leucine, which contributes to postprandial stimulation of muscle protein synthesis [20]. Dairy proteins, eggs, lean meats, fish, soy foods, and well-planned protein combinations can all be used within clinical or exercise-supportive dietary patterns, with food choice also accounting for affordability, cultural fit, gastrointestinal tolerance, cardiometabolic diet quality, and patient preference [20,38]. This broader food-based framing reflects that protein quality is not reducible to leucine content or isolated supplement use; the food matrix, saturated fat, sodium, fiber displacement, processing level, and dietary pattern all influence clinical interpretation.

Total daily intake remains the primary variable [41]. Even distribution across meals may help patients reach daily targets and may be useful when appetite is low, meal size is limited, or intake is concentrated in one large meal [33,40,41]. A protein-containing meal or supplement near resistance exercise can also be practical when it helps the patient meet daily targets, although rigid timing rules add complexity without clear evidence of clinical superiority [20,41]. Available evidence does not justify making protein timing more important than total intake, training consistency, or overall dietary quality [20,41]. A flexible approach is usually more suitable in obesity care: distribute protein in a way that supports satiety, tolerance, affordability, and the patient’s training schedule [20,38,41]. Overly prescriptive timing recommendations may reduce feasibility without adding clear clinical benefit, particularly in patients whose main barrier is simply achieving adequate intake consistently.

Plant-predominant dietary patterns require planning, not dismissal. They can achieve adequate protein intake when total dose, digestibility, amino-acid complementarity, and meal structure are considered [20,38]. The relevant issue is whether the diet can meet protein targets while preserving fiber intake, micronutrient adequacy, energy control, and adherence [20,38]. Sustainability is also relevant because dietary shifts toward more plant-forward patterns can reduce environmental pressures when they are nutritionally adequate and culturally feasible [42,43]. In this context, plant-based approaches are not inherently inferior, but they may require more deliberate planning to achieve adequate indispensable amino-acid intake and total protein dose within an energy-restricted diet [42,43,44].

### 4.5. Safety, Tolerability, and Clinical Controversies

Moderate increases in protein intake are generally acceptable for many adults without kidney disease, but this safety profile is not generalizable to all patients with obesity [20,37]. Renal status is the clearest example. In adults with chronic kidney disease who are at risk of progression, Kidney Disease: Improving Global Outcomes (KDIGO) advises avoiding high protein intake above 1.3 g/kg/day; in these patients, protein targets are individualized according to kidney function, albuminuria, diabetes status, nutritional risk, dialysis status when applicable, and nephrology or renal-dietetics guidance [37]. This caution is not a general prohibition against protein adequacy in all adults with obesity, but it argues against unsupervised escalation in patients with known or suspected kidney disease [37].

Tolerability can be just as limiting as physiology. Higher-protein dietary patterns may worsen early satiety, constipation, nausea, reflux, or food aversion in some patients, particularly during GLP-1 receptor agonist or tirzepatide therapy and after bariatric surgery [38,39,45,46]. In those contexts, smaller protein doses across meals, softer textures, oral nutrition supplements, hydration strategies, and dietitian involvement may be needed when appetite or intake capacity is limited [38,39,45,46]. Clinical interpretation therefore depends on how much protein is actually consumed, tolerated, and integrated into a nutritionally adequate diet, not only on the prescribed amount.

Several controversies follow from these constraints. First, high-protein diets are sometimes promoted as if they have a unique and universal fat-loss effect, whereas the more consistent evidence supports satiety in some patients and attenuation of lean-mass loss during caloric restriction [21,22]. Second, protein quality is not reducible to total grams alone because digestibility, indispensable amino-acid content, food matrix, processing, and whole-diet context influence interpretation [20,38]. Third, mechanistic concerns about excessive amino-acid exposure, including leucine and branched-chain amino acids, argue against assuming that more protein is always better or metabolically neutral [20]. The resulting conclusion is selective: higher-protein strategies can be useful when they improve satiety, support adherence, and help preserve lean tissue during weight loss, especially when paired with resistance or concurrent training [20,21]. Outside that context, their value depends on phenotype, energy deficit, protein source, achieved intake, safety, tolerance, and overall diet quality [32,37,38]. Thus, the evidence supports protein adequacy as a targeted supportive strategy rather than an unrestricted escalation of protein intake or a stand-alone obesity treatment.

### 4.6. Clinical Interpretation

High-protein diets are best characterized as supportive tools within obesity treatment, rather than stand-alone solutions [20,21]. Their strongest role is to improve the nutritional conditions under which weight loss occurs, particularly in patients at risk of lean-tissue loss or functional decline [21]. Accordingly, the rationale for higher-protein strategies is strongest in phenotype-specific contexts, including older adults, individuals with sarcopenic-obesity risk, patients with low baseline protein intake, and those undergoing rapid pharmacological or surgical weight loss [33,39,47,48]. Their effects on visceral adiposity, insulin sensitivity, inflammatory markers, and metabolic flexibility require cautious interpretation because these outcomes are likely influenced more directly by energy balance, fat-mass reduction, exercise training, and whole-diet quality than by protein intake alone [12,13,21,24]. Table 1 summarizes the main clinical uses, limitations, and interpretation of higher-protein nutrition in obesity. Causal attribution is limited because when a high-protein intervention is delivered alongside energy restriction, exercise, behavioral support, or supplementation, metabolic improvements cannot be attributed confidently to protein unless the study design isolates that effect.

This table summarizes clinical interpretation rather than fixed prescriptions. Protein targets are individualized according to weight reference, renal status, age, energy deficit, training status, appetite, gastrointestinal tolerance, dietary quality, and clinical goals. Abbreviations: g/kg, grams per kilogram.

This interpretation leads naturally to exercise. Protein can supply substrate and improve the nutritional environment for lean-tissue preservation, but training provides the mechanical and metabolic stimulus required to maintain or improve strength, cardiorespiratory fitness, and physical function [12,13,20]. Protein-focused nutrition is incomplete when functional preservation is a priority and no progressive exercise stimulus is provided.

## 5. Exercise Training in Obesity

Exercise training in obesity functions as a therapeutic stimulus for tissue remodeling, metabolic adaptation, and functional preservation, not only as a way to increase energy expenditure [12,13,18]. Exercise-induced weight loss may be modest when energy intake is not concurrently modified, whereas improvements in cardiorespiratory fitness, body composition, insulin sensitivity, blood pressure, strength, and physical performance can occur with smaller changes in scale weight [18,49,50]. For this reason, exercise outcomes in obesity include regional adiposity, fitness, muscle performance, symptoms, quality of life, adherence, and functional capacity, while avoiding the assumption that limited scale-weight change implies limited clinical value [12,13,18].

Recent consensus and systematic-review evidence support a multimodal approach to physical activity in adults with excess adiposity [12,13,18]. The 2024 American College of Sports Medicine consensus statement emphasizes that physical activity remains relevant across lifestyle, pharmacological, and bariatric treatment pathways, with exercise programs described as progressive, medically appropriate, inclusive, and adapted to symptoms, preferences, and barriers [18]. The EASO Physical Activity Working Group similarly frames structured exercise as a tool for fat-mass reduction, lean-mass preservation, physical fitness, quality of life, and long-term health, not only for weight reduction [12,13]. The central implementation question is which combination of stimuli can be performed consistently, progressed safely, and matched to the patient’s phenotype, symptoms, and goals, rather than which single modality ranks highest [12,13,18].

### 5.1. Resistance Training: Muscle Mass, Strength, and Functional Capacity

Resistance training provides the most direct exercise stimulus for skeletal-muscle loading, making it central when obesity treatment aims to preserve or improve strength, neuromuscular function, and physical reserve [15,18]. Its clinical value extends beyond hypertrophy. Mechanical tension, motor-unit recruitment, connective-tissue adaptation, improved coordination, and greater sensitivity of skeletal muscle to dietary amino acids may all contribute to functional improvement during weight management [20,51,52,53]. These adaptations are particularly relevant in adults with obesity because excess body mass increases the absolute load required for walking, stair climbing, rising from a chair, and balance recovery, while low relative strength can limit mobility even when absolute lean mass appears adequate [4,5,6]. However, the degree to which resistance training preserves measured lean mass during weight loss depends on training dose, progression, nutritional adequacy, adherence, baseline training status, and the method used to assess body composition.

Meta-analytic evidence supports resistance training as an obesity-relevant intervention even when total body weight changes are modest [15,16]. Resistance training can improve body-composition outcomes, including body-fat percentage and fat mass, and may contribute to favorable changes in visceral fat, although effects vary by population, comparator, training dose, and outcome method [15,16]. The endpoint extends beyond measured lean mass. In many adults with obesity, strength, power, balance, rate of force development, and task-specific function may be more clinically meaningful than small changes in appendicular lean mass on DXA [3,4,5,6]. This is especially relevant in older adults or patients with sarcopenic-obesity risk, in whom myosteatosis, inflammation, insulin resistance, inactivity, and anabolic resistance can reduce muscle quality despite apparently preserved fat-free mass [53]. Accordingly, resistance training is evaluated not only as a body-composition intervention but also for preservation or restoration of usable force production.

Resistance training may also contribute to metabolic health through mechanisms that are only partly reflected by weight change. Acute muscle contraction increases glucose uptake through insulin-independent pathways, while repeated training can improve insulin sensitivity, muscle oxidative capacity, and the function of a tissue compartment that is central to postprandial glucose disposal [49,54,55]. In adults with type 2 diabetes, overweight, or obesity, combined aerobic and resistance training has been associated with favorable body-composition and cardiometabolic outcomes [19,55]. Prescription remains progressive and technically safe: a practical starting point is two nonconsecutive sessions per week using major movement patterns and large muscle groups, progressing toward two to three weekly sessions as tolerance, confidence, and recovery improve [10,11,12,13,18]. Machines, free weights, resistance bands, body-weight movements, aquatic resistance, and functional tasks can all be appropriate when they permit adequate effort without worsening joint pain, fall risk, or discouragement [18,56]. The most effective resistance-training modality in routine care is often the one that allows repeated exposure, progressive overload, acceptable symptoms, and confidence rather than the one that appears optimal in a controlled trial.

### 5.2. Aerobic Training: Cardiorespiratory Fitness, Adiposity, and Metabolic Health

Aerobic training is the main exercise modality for improving cardiorespiratory fitness, increasing sustained energy expenditure, and enhancing whole-body oxidative capacity in adults with obesity [18,49,50]. Cardiorespiratory fitness is clinically important because low fitness is associated with impaired functional capacity and cardiometabolic risk, and improvements in fitness may occur even when body-weight reduction is limited [18,50]. Aerobic exercise therefore functions as a health and function intervention, not only as a method for producing an energy deficit [13,18]. Aerobic training may produce meaningful improvements in fitness or cardiometabolic risk even when compensatory changes in appetite, activity, or energy intake limit weight loss.

Dose still matters. A 2024 dose–response meta-analysis reported that aerobic exercise reduces body weight, waist circumference, and body-fat measures in adults, with larger effects generally requiring higher weekly volumes [50]. The ACSM consensus similarly emphasizes a dose–response relationship between moderate-intensity physical activity and adiposity outcomes, while recognizing that higher volumes may be needed for larger weight reduction or prevention of weight regain [18]. In clinical practice, the dose that can be repeated safely is more useful than an idealized target that cannot be sustained. Walking is accessible for many patients, but cycling, swimming, elliptical training, rowing, aquatic exercise, recumbent stepping, or arm ergometry may be preferable when knee osteoarthritis, low back pain, neuropathy, severe deconditioning, dyspnea, or very high body mass limits tolerance [12,13,18,56]. Thus, aerobic prescription distinguishes between the dose associated with measurable adiposity reduction and the dose that an individual patient can realistically accumulate without pain flares, injury, or disengagement.

Aerobic exercise is also relevant for visceral and ectopic fat, which are more closely linked to cardiometabolic risk than total body weight alone [1,3,14]. Network meta-analytic evidence indicates that multiple exercise modalities can reduce visceral adipose tissue in adults with overweight or obesity, with vigorous aerobic and interval-based approaches often ranking favorably; these rankings are comparative and indirect [14,57]. Exercise training may also improve blood pressure, insulin resistance, and intrahepatic fat in adults with overweight or obesity, but higher-intensity approaches require attention to symptoms, supervision, and musculoskeletal tolerance [49,58]. In metabolic dysfunction-associated steatotic liver disease, regular moderate- to vigorous-intensity aerobic exercise is supported for reducing hepatic steatosis and improving cardiometabolic risk, while resistance training remains a complementary modality [49,58].

### 5.3. Concurrent Training: An Integrated Strategy for Multidomain Obesity Care

Concurrent training combines resistance and aerobic exercise within the same program. Its rationale is not that it maximizes every individual adaptation, but that it addresses several obesity-relevant endpoints within one intervention: fat mass, visceral adiposity, cardiorespiratory fitness, insulin sensitivity, strength, muscle function, and physical capacity [17,19,55]. This integrative logic is particularly useful when treatment goals include fat reduction, cardiometabolic improvement, and preservation of function rather than a single performance or weight-loss endpoint [17,19].

The evidence requires careful interpretation. A network meta-analysis of 81 randomized controlled trials comparing exercise modalities in adults with overweight or obesity reported that combined training ranked favorably for several cardiometabolic outcomes, but rankings from indirect comparisons do not establish universal superiority [19]. The clinical argument is stronger when concurrent training is framed as a practical way to cover multiple domains. Patients with insulin resistance, type 2 diabetes, dyslipidemia, hypertension, low fitness, or sarcopenic-obesity risk may benefit from both aerobic and resistance stimuli because outcomes span cardiometabolic risk and muscular function [19,49,55]. Nevertheless, combined programs can also increase time demand, fatigue, logistical complexity, and adherence burden. These implementation costs influence translation of trial protocols into routine care.

Concerns about the so-called interference effect between endurance and resistance adaptations are also context-dependent [17,18]. The interference concept was classically described by Hickson, who reported attenuated strength development when strength and endurance training were performed concurrently compared with strength training alone [59]. Interference may matter for trained athletes seeking maximal strength or endurance performance, but most sedentary adults with obesity have substantial adaptive potential across both domains [17,18]. In clinical care, weekly dose, progressive overload, recovery, symptom control, and adherence usually matter more than theoretical optimization of session order [12,13,18]. Resistance and aerobic work can be performed on separate days when fatigue, schedule flexibility, or symptom management favors separation. When both modes are performed in the same session, sequence can follow the priority of the day, with resistance first when force output or technical quality is central and aerobic first when conditioning, enjoyment, or adherence is the immediate goal [12,13,18,60]. This flexible interpretation is more defensible than prescribing a single sequence, because the optimal order may differ according to the target outcome, patient preference, pain, fatigue, and available supervision.

A realistic concurrent-training program progresses across months. Many patients require an initial phase focused on movement tolerance, confidence, basic strength, and aerobic continuity before higher workloads are introduced [12,13,18]. This is especially relevant for patients with severe obesity, musculoskeletal pain, low exercise self-efficacy, prior negative experiences with exercise, or cardiometabolic comorbidities requiring monitoring [18,60]. Early success is therefore reflected by continuity, symptom tolerance, skill acquisition, and progression capacity, not only by immediate changes in body weight or exercise volume.

### 5.4. High-Intensity Interval Training: Potential Benefits and Clinical Cautions

High-intensity interval training alternates periods of vigorous effort with active or passive recovery and has been studied as a time-efficient strategy for improving cardiorespiratory fitness and cardiometabolic outcomes in adults with overweight or obesity [14,54,61]. The term HIIT covers widely different protocols, including differences in intensity, duration, work-to-rest ratio, total volume, modality, and supervision, so it does not represent a single standardized intervention [18,61]. Available evidence suggests that interval training can reduce body-fat percentage and some regional adiposity measures compared with nonexercise control, and some analyses report modest advantages over moderate-intensity continuous training for selected outcomes [54,61]. Network meta-analyses also suggest that HIIT can rank favorably for visceral adiposity, waist circumference, triglycerides, fasting glucose, and cardiorespiratory fitness in adults with overweight or obesity [14,54]. These findings are promising, but they are vulnerable to heterogeneity in protocol design, comparator intensity, supervision, dropout, and participant selection.

Those findings do not justify prescribing HIIT as universally superior to continuous aerobic training. The ACSM consensus concluded that HIIT does not appear superior to moderate-to-vigorous physical activity for regulation of excess body weight and adiposity, although it may be useful for selected patients when time efficiency, preference, supervision, and tolerance are favorable [18]. In this review, HIIT is presented as an optional component of a broader program rather than a replacement for resistance training, total weekly activity, or moderate-intensity aerobic exercise [12,13,18]. Clinical use requires screening, progression, and attention to musculoskeletal and cardiovascular risk [18,61]. Sedentary adults with obesity, patients with uncontrolled hypertension, unstable cardiovascular disease, severe joint pain, high fall risk, advanced diabetes complications, or very low exercise confidence may require a preparatory phase of low- to moderate-intensity training before vigorous intervals are attempted [12,13,18]. When HIIT is used, lower-impact modalities such as cycling, rowing, elliptical exercise, aquatic intervals, or incline walking may reduce orthopedic stress compared with running-based protocols [18,61]. Therefore, HIIT is treated as a phenotype- and preference-sensitive option rather than as a default intervention or a marker of superior program quality.

### 5.5. Feasibility, Safety, and Adherence in Adults with Obesity

The effectiveness of exercise prescription in obesity is constrained by adherence, accessibility, and tolerability as much as by physiological efficacy [18,60]. Common barriers include musculoskeletal pain, low baseline fitness, fatigue, breathlessness, weight stigma, limited access to safe facilities, cost, transport limitations, time constraints, embarrassment, caregiving or work demands, and previous negative experiences with exercise [18,60]. These barriers are clinical variables that shape prescription rather than simple failures of motivation. A program that is physiologically sound but incompatible with the patient’s pain, environment, finances, schedule, or confidence is unlikely to produce sustained benefit [18,60]. This is a major limitation when interpreting supervised exercise trials. Efficacy under structured conditions may overestimate what can be achieved without ongoing support, equipment access, behavioral counseling, or adaptation to daily constraints.

Safety screening is proportionate to clinical risk [18]. Many adults with obesity can begin low- to moderate-intensity activity without extensive testing, but patients with known cardiovascular disease, symptoms suggestive of unstable disease, severe hypertension, advanced diabetes complications, severe obstructive sleep apnea, major mobility impairment, or high fall risk may require medical assessment and supervised progression [18]. Clinicians and exercise professionals also monitor joint symptoms, dizziness, exertional chest discomfort, severe breathlessness, hypoglycemia risk in treated diabetes, pain flares, and signs of overuse injury [18]. Adherence can be improved by setting functional goals, progressing gradually, allowing patient choice of modality, providing supervision or follow-up, reducing weight-stigmatizing language, and emphasizing health outcomes beyond scale weight [12,13,18,60]. This approach may help sustain training when weight loss is slow but fitness, symptoms, or function are improving.

### 5.6. Exercise Prescription in Obesity: Practical FITT Considerations

Exercise prescription specifies frequency, intensity, time, type (FITT), as well as progression while remaining flexible enough to accommodate clinical phenotype and adherence barriers [18,56]. For many adults with obesity, a reasonable starting point is progressive aerobic activity on three or more days per week and resistance training on two nonconsecutive days per week, with gradual increases in duration, intensity, and movement complexity as symptoms and confidence improve [10,11,18]. The longer-term target may include at least 150 min per week of moderate-intensity aerobic activity, higher weekly volumes when adiposity reduction or weight-loss maintenance is a major goal, and two or more weekly muscle-strengthening sessions involving major muscle groups [10,11,18,50]. These targets are presented as progressive goals rather than immediate requirements, particularly for sedentary patients, patients with pain, and those with low exercise self-efficacy.

Intensity is individualized using tools that patients and clinicians can apply consistently, including rating of perceived exertion, talk test, heart-rate response, symptoms, and, when available, cardiopulmonary or field testing [18,62]. Moderate-intensity aerobic exercise usually allows sustained activity with increased breathing but preserved ability to speak, whereas vigorous intervals require greater caution and more deliberate recovery planning [61]. Resistance-training intensity can progress from low to moderate effort toward moderate to high effort using repetitions in reserve, perceived exertion, technical quality, and recovery rather than fixed load prescriptions alone [15,16]. Across modalities, progression prioritizes continuity before volume or intensity, especially in patients with pain, low fitness, severe obesity, or low exercise confidence [12,13,18]. Several weeks of movement familiarization, short bouts, low-impact aerobic work, and basic resistance exercises may be necessary before conventional guideline volumes are realistic [12,13,18]. Supervised or partially supervised programs may be particularly useful at the start because they can improve technique, confidence, symptom interpretation, and progression decisions [18,60]. Table 2 summarizes the main clinical roles, practical uses, and cautions for exercise modalities in obesity care. A clinically useful FITT prescription therefore includes the target dose, starting dose, progression criteria, symptom thresholds, and fallback options when pain, fatigue, or adherence barriers emerge.

Modalities are not mutually exclusive and are adapted to symptoms, access, baseline fitness, comorbidities, safety, and adherence. The table is intended as a clinical framework, not as a ranked hierarchy of exercise efficacy. Abbreviations: HIIT, high-intensity interval training; min/week, minutes per week.

## 6. Complementary Effects of Higher-Protein Diets and Exercise in Obesity

**Figure 2:**
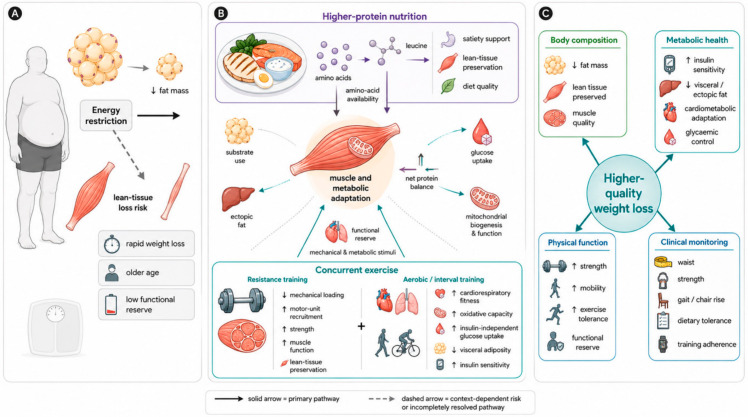
Conceptual framework linking higher-protein nutrition and concurrent exercise to higher-quality weight loss in adults with obesity. Panel (**A**) shows that energy restriction is usually required for clinically meaningful fat-mass reduction, but may also increase the risk of lean-tissue loss, particularly during rapid weight loss, older age, or low functional reserve. Panel (**B**) illustrates the complementary biological roles of higher-protein nutrition and concurrent exercise. Protein-rich dietary patterns may support amino-acid availability, satiety, diet quality, net protein balance, and lean-tissue preservation, while resistance training provides mechanical loading and neuromuscular stimuli relevant to strength and muscle function. Aerobic and interval-based training provide complementary metabolic stimuli, including improvements in cardiorespiratory fitness, oxidative capacity, insulin-independent glucose uptake, insulin sensitivity, and visceral adiposity reduction. Panel (**C**) summarizes the clinical interpretation of higher-quality weight loss as a multidomain outcome that includes body composition, metabolic health, physical function, and clinical monitoring rather than scale-weight reduction alone. Solid arrows indicate primary conceptual pathways; dashed arrows indicate context-dependent risks or incompletely resolved pathways. The figure is intended as an integrative clinical–physiological model rather than evidence of a universal synergistic effect across all obesity phenotypes [3,4,5,6,12,13,14,15,16,17,18,20,21,49,50,51,52,53,64,65,66]. Abbreviations: ↑ Increase; ↓ decrease.

The rationale for combining higher-protein nutrition with structured exercise in obesity is not that the combination reliably produces greater scale-weight loss than either strategy alone. Its stronger rationale is that energy restriction, protein adequacy, and exercise training influence different dimensions of weight-loss quality [3,21]. Energy restriction is usually needed for clinically meaningful fat loss, but it can also increase the risk of fat-free mass loss, especially in older adults, patients with sarcopenic-obesity risk, and individuals undergoing rapid weight reduction [4,5,6,21]. Protein availability may improve the nutritional environment for lean-tissue preservation, whereas resistance and aerobic exercise provide the mechanical and metabolic stimuli needed to maintain or improve muscle function, fitness, and cardiometabolic adaptation [20,51,52,53]. For that reason, this review uses the term complementary deliberately. The available evidence supports a biologically coherent and clinically useful pairing, but it does not consistently demonstrate synergy in the strict statistical sense. This complementary framework is summarized in Figure 2, which illustrates how energy restriction, higher-protein nutrition, and concurrent exercise may influence different but interacting dimensions of weight-loss quality. In this model, energy restriction primarily supports fat-mass reduction but may also increase lean-tissue loss risk in vulnerable contexts, whereas higher-protein nutrition and exercise provide nutritional, mechanical, and metabolic stimuli that may help preserve muscle-related function and improve cardiometabolic adaptation.

### 6.1. Biological Rationale for Combining Protein and Exercise

Skeletal muscle adapts to the combined influence of contractile activity, amino-acid availability, energy status, insulin signaling, inflammation, and recovery [20,52,53,67]. Resistance exercise increases the sensitivity of skeletal muscle to amino acids and stimulates myofibrillar remodeling, while protein ingestion provides indispensable amino acids required for post-exercise muscle protein synthesis [20,51,52,53]. During caloric restriction, this interaction becomes clinically relevant because the same energy deficit that supports fat loss may also reduce the anabolic environment needed for lean-tissue preservation [21,68]. The mechanistic rationale is plausible but proportional to the evidence: mechanical loading activates pathways involved in muscle remodeling, amino-acid availability supports translational responses linked to muscle protein synthesis, and leucine contributes to this response as one component of protein quality rather than as an isolated determinant of adaptation [67,69]. This is an important distinction because mechanistic plausibility can easily be overstated. Acute stimulation of muscle protein synthesis does not automatically translate into preserved muscle quality, higher strength, or better mobility during a prolonged weight-loss intervention.

Obesity can complicate this physiology. Chronic low-grade inflammation, insulin resistance, myosteatosis, mitochondrial dysfunction, physical inactivity, and aging-related anabolic resistance may reduce muscle quality and attenuate adaptive responses to nutrition or exercise in some phenotypes [5,6,53,70]. These mechanisms help explain why some adults with obesity may have normal or elevated lean mass while still showing low relative strength, poor mobility, or reduced physical reserve [4,5,6]. Aerobic and interval-based exercise add a different stimulus by challenging cardiorespiratory and oxidative systems: muscle contraction increases glucose uptake through insulin-independent mechanisms, and repeated aerobic training can improve oxidative capacity, mitochondrial adaptation, vascular function, and substrate utilization [64,65,66]. Protein may support the structural side of adaptation by helping preserve lean tissue during energy restriction, but exercise remains the principal stimulus for improving fitness and oxidative metabolism [64,65,66]. Thus, protein is not a substitute for training. It is better understood as one condition that may help the patient respond to training and tolerate weight loss with less functional compromise.

### 6.2. From Lean Tissue to Usable Function

During weight loss, interpretation extends beyond whether muscle protein synthesis can be stimulated after a meal or training session. The harder clinical question is whether muscle-related function can be preserved over weeks and months while body mass is declining [3,21]. Enhanced protein intake can attenuate muscle-mass loss during weight-loss interventions in adults with overweight or obesity, but effects on strength and physical function are less consistent [21]. Lean-mass preservation is clinically incomplete if mobility, strength, power, fatigue resistance, or exercise tolerance do not improve or at least remain stable [3,4,5,6]. Protein can improve the nutritional environment for lean-tissue retention, but it does not substitute for progressive mechanical loading [21,53]. Resistance training is therefore difficult to replace when functional preservation is a priority.

The direct evidence is promising but not uniform. Trials in adults with obesity and related populations suggest that combined protein and exercise strategies can improve body composition and selected functional or metabolic outcomes, although responses vary according to protein target, achieved intake, supplement composition, training adherence, supervision, energy deficit, and baseline phenotype [21,23,24,68,71,72]. Evidence from older adults supports the plausibility of combining protein intake with resistance training for appendicular lean mass and strength, but transfer to obesity care remains cautious because trial populations and intervention contexts differ [73,74,75]. This caution matters because an intervention tested in relatively healthy older adults, rehabilitation settings, or tightly supervised trials may not perform the same way in adults with severe obesity, pain, low fitness, medication-related appetite suppression, or limited access to exercise support.

Obesity-specific evidence is therefore more conditional than the biological rationale might suggest. Some trials show preservation of appendicular muscle mass or favorable within-group changes, whereas others do not demonstrate a clear interaction between high protein and resistance exercise [71,72]. That pattern does not invalidate the combined approach; it narrows the claim. Higher-protein nutrition plus exercise is best presented as a plausible and partly supported strategy for lean-tissue preservation and functional protection, not as a uniform guarantee of superior outcomes across all obesity phenotypes [21,23,24,68].

### 6.3. Body Composition and Regional Adiposity

The main body-composition question is whether protein adequacy and exercise improve the proportion of weight lost as fat mass rather than lean tissue [21]. Enhanced protein intake can attenuate muscle-mass loss during weight reduction, while exercise training contributes to fat loss, fitness, and functional adaptation [21]. Concurrent training is useful in this context because it combines two exercise stimuli that target different components of weight-loss quality: resistance training provides the most direct stimulus for preserving or increasing strength and lean tissue, while aerobic or interval-based training contributes more consistently to cardiorespiratory fitness, regional adiposity reduction, and cardiometabolic adaptation [17,49,58]. Protein may reinforce the lean-tissue side of the intervention, but the fat-mass side still depends on the broader energy and activity context [18,50]. Accordingly, reductions in visceral or ectopic fat are not attributable to protein intake alone when the intervention also includes energy restriction, aerobic exercise, or substantial weight loss.

Measurement complicates interpretation. DXA-derived lean mass, appendicular lean mass, muscle cross-sectional area, strength, and physical performance are related but not interchangeable outcomes [3,4]. A combined intervention that preserves lean mass but fails to preserve strength, gait capacity, or chair-rise performance may have limited functional significance [4,5,6]. Conversely, strength or mobility may improve even when measured lean mass changes little, especially early in training when neural adaptation, movement confidence, and task-specific practice contribute to performance gains [15,16]. No single combined protocol can be treated as optimal across all contexts because protein dose, weight denominator, energy deficit, modality, supervision, adherence, and body-composition method all influence interpretation [21,23,24,68]. Tissue outcomes and functional outcomes are therefore reported together rather than allowing either one to stand in for the other.

### 6.4. Metabolic Health, Inflammatory Markers, and Metabolic Flexibility

The metabolic effects of higher-protein nutrition plus exercise are likely mediated through several overlapping pathways, including reduction in fat mass and visceral adiposity, improved cardiorespiratory fitness, enhanced skeletal-muscle glucose uptake, increased oxidative capacity, and preservation of metabolically active lean tissue [49,55]. In randomized trials of middle-aged adults with obesity, high-protein diet combined with exercise has been associated with favorable changes in body composition and selected functional outcomes; related analyses have also reported improvements in selected cardiometabolic and inflammatory markers over short follow-up periods [23,24]. These findings are encouraging, but they do not establish protein intake as the independent driver of metabolic improvement. Exercise is probably the dominant active stimulus for several adaptations: improvements in insulin sensitivity may occur through contraction-mediated glucose uptake and training-induced increases in oxidative and glucose-transport capacity, resistance training may preserve or increase the contractile tissue compartment available for glucose disposal, and aerobic training may improve mitochondrial and vascular adaptations that support substrate oxidation [64,65,66]. Protein supports these adaptations indirectly when it helps preserve lean tissue during energy restriction, but it is not the primary metabolic intervention in isolation [20,21]. Attribution is difficult because combined interventions usually change several variables at once: energy intake, body weight, fat mass, training exposure, fitness, and sometimes dietary quality.

Metabolic flexibility and inflammatory markers require additional caution. Metabolic flexibility is a useful integrative concept, but it remains undermeasured in clinical trials of combined protein and exercise interventions [64]. Whether higher-protein diets plus concurrent training restore metabolic flexibility beyond the effects of weight loss and exercise remains uncertain [64]. Inflammatory outcomes are similarly difficult to attribute. Markers such as *C*-reactive protein may improve when adiposity decreases and physical activity increases, but the independent contribution of protein intake is less clear [24,49]. When combined-intervention trials report changes in inflammatory markers, those effects may reflect fat-mass loss, improved fitness, or the broader lifestyle intervention rather than protein-specific effects [23,24,68]. A balanced interpretation is that protein may help protect the muscular substrate through which exercise exerts part of its metabolic effect, rather than acting as a stand-alone metabolic therapy.

### 6.5. Liver Health and Metabolic Dysfunction-Associated Steatotic Liver Disease

Liver health provides an additional example of why obesity interventions should be interpreted through metabolic and functional outcomes rather than scale weight alone. Metabolic dysfunction-associated steatotic liver disease (MASLD) is closely linked to obesity, insulin resistance, visceral adiposity, type 2 diabetes, and metabolic syndrome, and it can coexist with low muscle mass, poor muscle quality, or sarcopenic obesity in higher-risk phenotypes [31,76]. Exercise training is clinically relevant in this context because aerobic, resistance, and combined exercise can reduce hepatic steatosis and improve cardiometabolic risk factors, even when weight loss is modest [49,58].

Protein intake may also be relevant to liver health, but the interpretation should remain cautious and diet-pattern specific. Low protein intake or protein insufficiency has been discussed as a potential contributor to hepatic triglyceride accumulation and impaired metabolic adaptation, whereas higher-protein dietary patterns may support satiety, weight management, insulin sensitivity, and lean-mass preservation in some patients with obesity or MASLD [77,78,79]. However, this does not mean that higher protein intake should be treated as a stand-alone therapy for MASLD or that unrestricted protein escalation is appropriate for all patients. The hepatic relevance of protein depends on total energy intake, weight loss, protein source, overall diet quality, fiber intake, cardiometabolic risk, renal status, and exercise exposure [21,38,77,78].

From the perspective of this review, MASLD reinforces the need for integrated rather than macronutrient-isolated care. In adults with obesity and suspected or confirmed MASLD, protein adequacy may be most clinically meaningful when it supports preservation of muscle-related function during weight loss, while concurrent training provides complementary stimuli for cardiorespiratory fitness, insulin sensitivity, hepatic fat reduction, and physical function [49,58]. Evidence directly testing higher-protein nutrition combined with concurrent exercise specifically in MASLD remains limited; therefore, the current role of MASLD in this review is to illustrate a metabolically vulnerable phenotype in which body composition, liver health, and physical function should be monitored together.

### 6.6. High-Risk Phenotypes, Incretin-Based Pharmacotherapy, and Rapid Weight Loss

The main clinical rationale for combining protein adequacy with exercise is preservation or improvement of usable physical function during fat loss [21,68]. In some trials, exercise combined with a high-protein diet has improved muscle power, aerobic capacity, and functional physical performance more than control conditions in adults with obesity [23]. These outcomes matter because chair-rise ability, gait capacity, stair climbing, fatigue resistance, balance, and exercise tolerance are often more meaningful to patients than small differences in total weight loss [4,5,6]. Older adults and patients with sarcopenic-obesity risk deserve particular attention because they may begin treatment with reduced muscle quality, anabolic resistance, low physical activity, pain, or limited reserve [4,5,6,53,70]. Individuals undergoing rapid or high magnitude weight loss, including pharmacologically or surgically induced weight loss, represent another clinically important group because loss of body weight may include reductions in both fat mass and lean soft tissue [39,48]. In these patients, protein adequacy and resistance training are considered together rather than sequentially: protein supports the nutritional substrate for adaptation, while training determines whether preserved tissue is challenged to become stronger and more functional [21,53].

A related concern applies to rapid treatment-induced weight loss, including GLP-1 receptor agonists, dual GIP/GLP-1 receptor agonists such as tirzepatide, and bariatric surgery [39]. Major obesity pharmacotherapy trials have shown clinically substantial weight loss, and available body-composition substudies indicate that most weight lost is fat mass, but that lean soft tissue also declines during treatment [7,8]. In the STEP 1 DXA substudy, semaglutide 2.4 mg reduced total fat mass and visceral fat mass, while lean body mass also decreased in absolute terms, resulting in an improved lean-body-mass-to-fat-mass ratio [80]. In the SURMOUNT-1 DXA substudy, tirzepatide produced large reductions in body weight, fat mass, and visceral fat mass, but lean mass also declined; the authors reported improved body composition overall, yet functional measures such as strength, muscle quality, and physical performance were not comprehensively assessed [9]. These findings should not be interpreted as evidence that pharmacological weight loss causes clinically harmful muscle wasting, nor as proof that lean-mass reduction is functionally irrelevant. The clinical meaning of lean soft-tissue loss depends on baseline phenotype, age, magnitude and rate of weight loss, dietary intake, resistance-training exposure, muscle quality, strength, and physical trajectory [3,4,7,8,9].

Current RCTs of incretin-based pharmacotherapy were primarily designed to assess weight loss, cardiometabolic efficacy, and safety, not to test whether higher-protein nutrition or concurrent training mitigates lean-mass loss [81,82,83]. Dietary intake, achieved protein intake, resistance-training exposure, aerobic fitness, muscle strength, myosteatosis, and objective physical performance have generally been undercharacterized relative to total weight loss and fat-mass outcomes [81,82,83]. Direct randomized evidence testing high-protein concurrent training during incretin-based pharmacotherapy remains limited. Until stronger evidence is available, monitoring protein adequacy, resistance-training participation, aerobic conditioning, and functional trajectory in higher-risk patients is more consistent with the evidence than assuming that medication-induced weight loss preserves function [38,47,48,84]. This pairing is not a guarantee, but it is clinically rational because the consequences of losing function in these groups are higher. Table 3 summarizes selected RCTs and body-composition analyses that are relevant to the interpretation of lean soft-tissue change during incretin-associated weight loss, with emphasis on limitations directly related to the scope of this review.

Overall, higher-protein nutrition and exercise are best characterized as a combined strategy for improving the composition and functional meaning of weight loss in obesity. The clearest support relates to lean-tissue preservation and muscle-related outcomes when protein adequacy is paired with resistance training; effects on visceral fat, insulin sensitivity, inflammatory markers, and metabolic flexibility are shaped more strongly by exercise modality, total fat loss, energy balance, and the broader lifestyle context [19,55,57,58,68].

## 7. Clinical Applications and Practical Prescription

Clinical application begins with phenotype rather than with a fixed protocol. Adults with obesity differ in age, fat distribution, sarcopenic-obesity risk, cardiometabolic disease, medication exposure, renal status, pain, baseline fitness, access to care, and readiness for behavior change [1,5,6]. Higher-protein nutrition and concurrent exercise are therefore best used as adaptable clinical tools, not as a uniform prescription applied to all patients [20,21,85]. The clinical goal is to improve the composition and functional meaning of weight loss: reducing harmful adiposity while preserving or improving muscle-related function, cardiorespiratory fitness, metabolic health, and physical capacity [3,4,12,13]. The same intervention can therefore be appropriate, insufficient, or poorly tolerated depending on the patient’s starting point. A higher protein target and concurrent training plan therefore function as a clinical hypothesis to monitor, not as an automatically successful prescription.

This approach also changes what is monitored. Scale weight remains useful, but it is insufficient when the intervention aims to protect function and tissue quality [4]. Waist circumference, dietary intake, protein tolerance, strength, mobility, exercise tolerance, adverse symptoms, and patient-centered functional goals may provide a more complete view of whether treatment is improving health rather than simply reducing body mass [3,4,5,6]. Table 4 outlines phenotype-sensitive priorities for adapting protein-supported concurrent training in selected clinical contexts. The table is intended to guide clinical reasoning, not to replace individualized assessment or multidisciplinary care when comorbidity burden, renal risk, medication effects, or mobility limitations are substantial.

### 7.1. Phenotypes That May Require Closer Attention

Patients with sarcopenic obesity or low functional reserve are a priority group because excess adiposity and impaired muscle function jointly increase vulnerability [5,6]. In these patients, treatment extends beyond fat loss. It also addresses strength, gait, balance, fatigue resistance, confidence with movement, and preservation of independence [4,5,6]. Concurrent training plus adequate protein aligns with that goal because it combines muscle loading, aerobic conditioning, and amino-acid availability, although the intervention still needs to be adapted to pain, baseline function, safety, and adherence [20,21].

Older adults with obesity require a similar but not identical emphasis. Aging increases anabolic resistance, frailty risk, sarcopenia risk, and the likelihood that intentional weight loss will include clinically relevant lean-tissue loss [33,40,53]. In this group, maximal weight reduction is rarely the only meaningful target. Improvements in mobility, strength, central adiposity, glycemic control, symptoms, and day-to-day function may be more important than the largest possible change in body weight [4,5,6]. When feasible, chair-rise ability, gait speed, grip strength, balance, falls history, fatigue, and activities of daily living are tracked alongside weight and waist measures [4,5,6]. This interpretation does not make weight loss unimportant in older adults. Rather, it means that the acceptable trade-off between fat loss, lean-tissue change, symptoms, and independence is narrower.

Adults receiving GLP-1 receptor agonists or tirzepatide also deserve closer nutritional and functional monitoring. These therapies can produce substantial reductions in body weight and fat mass, while body-composition analyses show that lean mass may also decline during treatment [7,8,9]. That decline is not automatically pathological muscle wasting, but it is clinically relevant in patients who are older, have sarcopenic-obesity risk, report low intake, or begin treatment with low strength or limited mobility [7,8,9]. In these cases, early attention to protein adequacy, resistance training, aerobic conditioning, and functional trajectory is more defensible than assuming that pharmacological weight loss will preserve physical capacity [38,47,48,84].

**Figure 3:**
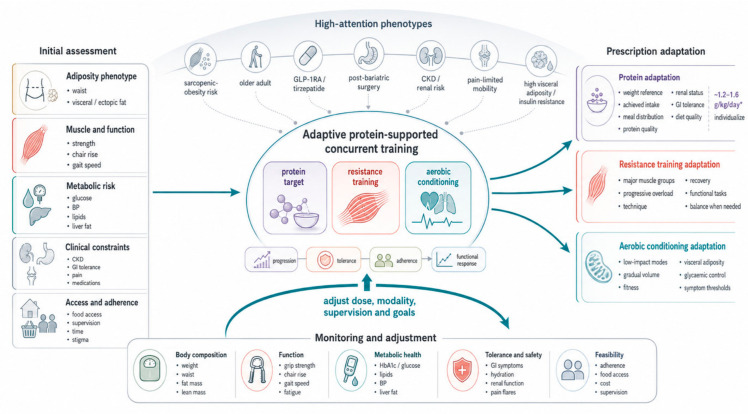
Phenotype-sensitive framework for adapting protein-supported concurrent training in obesity care. The figure illustrates an adaptive clinical pathway in which initial assessment informs individualized nutrition and exercise prescription, and monitoring data guide iterative adjustment. Initial assessment should include adiposity phenotype, muscle and functional status, metabolic risk, clinical constraints, and access or adherence barriers. High-attention phenotypes include sarcopenic-obesity risk, older age, incretin-associated appetite suppression or rapid weight loss, post-bariatric anatomy, chronic kidney disease or renal-risk profiles, pain-limited mobility, and high visceral adiposity or insulin resistance. Protein adaptation should consider the weight reference used to calculate intake, achieved intake, meal distribution, protein quality, renal status, gastrointestinal tolerance, and whole-diet quality; a pragmatic range of approximately 1.2–1.6 g/kg/day may be considered for many adults undergoing intentional weight loss, but should not be treated as a universal target. Resistance-training adaptation should emphasize major muscle groups, progressive overload, technique, recovery, functional tasks, and balance when needed, whereas aerobic conditioning should be adapted according to impact tolerance, gradual volume progression, fitness, visceral adiposity, glycemic control, and symptom thresholds. Monitoring should extend beyond scale weight to include waist circumference, body composition when feasible, strength, chair-rise performance, gait speed, fatigue, metabolic markers, renal function when clinically indicated, gastrointestinal symptoms, pain flares, adherence, food access, cost, and supervision needs. The framework is intended to support clinical reasoning and follow-up, not to replace individualized assessment or multidisciplinary care [4,5,6,7,8,9,10,11,12,13,15,16,17,18,19,32,37,38,39,41,45,46,47,48,49,55,58,62,63,84,90,91]. Abbreviations: BP, blood pressure; CKD, chronic kidney disease; GI, gastrointestinal; GLP-1RA, glucagon-like peptide-1 receptor agonist; HbA1c, glycated hemoglobin. *, the range depends on the weight reference, renal status, tolerance, energy deficit, training status, and clinical context.

Patients with insulin resistance, type 2 diabetes, metabolic dysfunction-associated steatotic liver disease, or high visceral adiposity may benefit from a concurrent-training framework because aerobic and resistance exercise act through complementary pathways [19,55,58]. For these phenotypes, clinical targets extend beyond scale weight to include waist circumference, blood pressure, glycemic indices, lipids, cardiorespiratory fitness, functional capacity, and, when clinically available, intrahepatic or visceral fat measures [49,55,58]. These phenotype-specific considerations can be translated into an adaptive clinical pathway rather than a fixed prescription. Figure 3 summarizes this approach by linking initial assessment, high-attention phenotypes, protein and exercise adaptation, monitoring, and iterative adjustment of dose, modality, supervision, and goals.

### 7.2. Protein Targets by Clinical Context

Protein targets in obesity are individualized because the denominator matters. Actual body weight can overestimate requirements in severe obesity, whereas ideal body weight may underestimate needs during energy restriction, active training, sarcopenic-obesity risk, or rapid treatment-induced weight loss [32,39]. Adjusted body weight, target body weight, or estimated fat-free mass may be more clinically useful than actual body weight when prescribing protein in adults with obesity [32]. For many adults undergoing intentional weight loss, approximately 1.2–1.6 g/kg/day using an appropriate weight reference can be used as a pragmatic working range, but not as a universal requirement [20,21,32]. A useful prescription therefore states both the numerical target and the weight reference used to calculate it; otherwise, the recommendation is difficult to interpret or reproduce.

Training status modifies interpretation. Sport-nutrition guidance often identifies 1.4–2.0 g/kg/day as sufficient for many exercising individuals, but translation into clinical obesity care remains cautious [20]. A physically active adult with obesity, preserved renal function, good tolerance, and progressive resistance training may justify a higher target than a sedentary patient with nausea, food insecurity, CKD risk, or poor intake capacity [32,37,38]. The prescription therefore reflects achieved intake, safety, tolerance, dietary quality, and the purpose of the intervention rather than a single idealized number. A lower target that is consistently achieved within a good diet may be more useful than a higher target that worsens symptoms or displaces other foods.

Older adults and patients with sarcopenic-obesity risk may need closer attention to protein quality, distribution, and per-meal dose because anabolic resistance can blunt the muscle protein synthetic response to feeding [33,40]. Protein intakes above the adult RDA are commonly recommended in older adults, with higher ranges considered in illness, frailty risk, or active rehabilitation when not contraindicated [33,40]. In adults with obesity, however, the selected weight denominator, renal function, appetite, comorbidities, and training plan guide the final target [32,37].

Patients using GLP-1 receptor agonists or tirzepatide may require planned protein distribution because early satiety, nausea, reduced intake, constipation, and food aversions can make spontaneous protein intake insufficient [38]. The clinical task is practical: assess actual intake, meal frequency, gastrointestinal tolerance, hydration, micronutrient adequacy, and capacity to perform resistance exercise [38,47,48,84]. Medication-induced weight loss does not necessarily preserve function without monitoring [38,47,48,84].

Post-bariatric patients often receive commonly cited targets such as at least 60–80 g/day or approximately 1.1–1.5 g/kg ideal body weight/day, with individualization by procedure, phase of recovery, intake capacity, tolerance, clinical follow-up, and evolving guidance [39,45,46]. Smaller and more frequent protein doses, oral nutrition supplements, texture modification, hydration planning, and micronutrient monitoring may be necessary when intake capacity is limited [39,45,46]. Renal status remains a separate safety consideration: KDIGO advises avoiding high protein intake above 1.3 g/kg/day in adults with chronic kidney disease who are at risk of progression, and CKD-related targets are individualized with nephrology or renal-dietetics guidance when appropriate [37]. This makes post-bariatric and renal-risk contexts poor candidates for generic high-protein advice without follow-up.

### 7.3. Exercise Prescription: From FITT to Feasible Progression

Exercise prescription specifies frequency, intensity, time, type, progression, and safety monitoring, with these elements functioning as a clinical framework rather than as a rigid template [62]. Preparticipation screening considers current activity level, symptoms, known cardiovascular, metabolic or renal disease, and desired exercise intensity because these variables modify exercise-related risk [62,63]. For many sedentary adults with obesity, the safest initial strategy is gradual progression from low or moderate intensity rather than abrupt exposure to high-volume or high-intensity training [18,62]. The first prescription is most useful when it can be repeated the following week, rather than only satisfying a guideline on paper.

A practical starting point is to combine progressive aerobic activity with resistance training on at least two nonconsecutive days per week, increasing duration, intensity, and complexity as symptoms, confidence, and recovery allow [10,11,12,13,18]. Aerobic work may begin with short, repeatable bouts of walking, cycling, aquatic exercise, elliptical training, rowing, or recumbent stepping when orthopedic or respiratory symptoms limit tolerance [18,56]. Resistance training emphasizes major movement patterns, large muscle groups, technical quality, and progressive effort, with machines, bands, free weights, body-weight tasks, aquatic resistance, or functional movements selected according to access and safety [15,16,18]. HIIT can be considered later for selected patients who tolerate vigorous effort, although it does not replace moderate-intensity activity, resistance training, or total weekly movement volume [18,61]. This sequencing keeps the emphasis on building capacity first; intensity can be added when it improves the program rather than when it simply makes the program appear more ambitious.

The order of aerobic and resistance training is guided by the patient’s main limitation and the purpose of the session. If strength, balance, or technical learning is the priority, resistance training may be placed earlier; if conditioning, enjoyment, or adherence is the immediate barrier, aerobic exercise may come first [12,13,18]. Separate-day programming can help when fatigue or pain limits same-session quality, whereas same-session concurrent training may be more realistic for patients with limited time or transport access [12,13,18,60]. In most clinical settings, consistency, progression, symptom control, and adherence are more important than theoretically optimal sequencing [18,60].

### 7.4. Protein Timing, Distribution, and Diet Quality

Protein timing is secondary to total daily intake, protein quality, dietary adequacy, and training consistency [20,41]. A protein-containing meal or supplement near resistance exercise may help patients reach daily targets, but rigid timing rules add burden without clear evidence of superiority when total intake is adequate [20,41]. Distribution becomes more relevant when appetite is low, meal size is limited, or early satiety is induced by incretin therapy or bariatric surgery [38,45,46]. In this context, timing functions as an adherence tool rather than as a central mechanism of the intervention.

Protein quality is interpreted within the whole dietary pattern. Dairy proteins, eggs, lean meats, fish, soy foods, and carefully planned plant-based combinations can support indispensable amino-acid intake, while fiber-rich foods, fruits, vegetables, legumes, whole grains, and unsaturated fats remain important for cardiometabolic health [20,38]. In patients with food insecurity or limited access, useful plans emphasize affordable protein sources and realistic preparation methods rather than reliance on supplements [38,60]. A protein plan that is unaffordable, culturally mismatched, or dependent on supplements is unlikely to be durable, even when it is nutritionally plausible.

### 7.5. Implementation, Monitoring, and Safety

Implementation determines whether the prescription becomes treatment or merely advice. Cost can limit access to high-quality protein foods, supplements, resistance-training facilities, supervised exercise, transport, and clinical monitoring [38,60]. Clinicians may consider lower-cost protein options, home-based resistance tools, community programs, telehealth follow-up, and patient-selected exercise modes when these improve feasibility [60,90]. This implementation step is integral to the intervention. In obesity care, feasibility is part of the intervention, not an administrative detail. Multidisciplinary care is often needed when obesity management involves complex nutrition targets, exercise progression, pharmacotherapy, bariatric history, renal-risk profiles, pain-limited mobility, or functional decline; coordinated input from physicians, dietitians, exercise professionals, physiotherapists, psychologists, and bariatric or renal specialists can help align safety, tolerability, adherence, and clinical goals [18,60,85].

Tolerability can determine success as much as physiology. Higher-protein dietary patterns may worsen early satiety, constipation, nausea, reflux, or food aversions, especially during incretin therapy or after bariatric surgery [38,39,45,46]. Smaller protein doses across meals, softer textures, liquid supplements, hydration planning, and symptom-targeted nutrition strategies can help when intake capacity is limited [38,39,45,46]. Protein targets are adjusted if they worsen gastrointestinal symptoms or displace other essential components of the diet [38].

Exercise barriers are clinical variables rather than motivational failures. Pain, dyspnea, low fitness, fear of injury, embarrassment, weight stigma, lack of safe spaces, time constraints, caregiving responsibilities, and previous unsuccessful attempts can all reduce adherence [60]. Supervision, gradual progression, symptom-contingent modifications, non-stigmatizing communication, and patient-selected modalities can improve feasibility while preserving the physiological targets of concurrent training [60,90].

Monitoring matches the reason for prescribing the combined intervention. Body weight and waist circumference remain useful, but interpretation also includes dietary intake, protein tolerance, renal function when clinically indicated, medication side effects, strength, mobility, cardiorespiratory tolerance, fatigue, adverse symptoms, and patient-centered goals [18,37,38]. Body composition is measured when feasible, but DXA-derived lean mass is not equivalent to muscle quality or physical function [3,4]. A clinically meaningful endpoint is sustained improvement in adiposity, metabolic health, and physical capacity, not short-term weight loss alone [3,4,5,6]. If monitoring shows weight loss with worsening fatigue, declining strength, poor intake, or reduced mobility, the intervention warrants reconsideration even when the scale is moving in the desired direction.

## 8. Current Gaps and Research Priorities

The evidence base for higher-protein diets combined with exercise in obesity is clinically relevant, but it remains too heterogeneous to support a single standardized prescription across patient phenotypes [21,23,24,68]. Protein interventions differ in how dose is defined: grams per day, grams per kilogram of actual body weight, adjusted or target body weight, percentage of energy intake, or supplemental protein added to habitual intake [20,21,32]. Exercise interventions vary in modality, intensity, weekly volume, supervision, progression, adherence support, and comparator condition [17,18]. This heterogeneity limits interpretation because observed effects may reflect protein dose, achieved intake, energy restriction, resistance-training exposure, aerobic volume, behavioral support, or the interaction among these components. Future studies would benefit from standardized reporting of prescribed and achieved protein intake, protein source, weight denominator, energy deficit, meal distribution, resistance-training load, aerobic-training dose, supervision, adherence, and adverse events [21,91].

Duration is a second limitation. Many nutrition-exercise trials in adults with overweight or obesity are long enough to detect early changes in body weight or body composition, but not long enough to determine whether lean-tissue preservation, strength, mobility, cardiorespiratory fitness, or weight-loss maintenance are durable [21,23,24,68,91]. This matters because the clinical value of a combined intervention depends not only on initial fat loss, but also on whether patients maintain function, activity, and metabolic improvement over time [3,4,91]. Longer trials are needed with maintenance phases, repeated functional assessment, adverse-event reporting, and monitoring of whether prescribed protein and exercise targets are actually achieved [90,91,92]. A short trial can show that a strategy is biologically active; however, it does not establish that the same strategy is livable, safe, or function-preserving over the period in which obesity care usually occurs.

Outcome selection also needs refinement. Body weight, BMI, and total fat mass are insufficient when the purpose of treatment is to improve the quality of weight loss [3]. Future trials would be strengthened by assessing fat mass, lean mass, appendicular lean mass, muscle cross-sectional area, myosteatosis, visceral adiposity, ectopic fat, strength, power, gait speed, chair-rise performance, stair-climb capacity, aerobic capacity, fatigue, quality of life, and patient-reported mobility when feasible [93,94,95,96,97,98]. These outcomes are not interchangeable. A trial that preserves DXA-derived lean mass but does not measure strength or mobility cannot determine whether the preserved tissue translated into function [3,4,5,6]. Conversely, functional improvement with minimal change in measured lean mass may still be clinically meaningful, particularly early in resistance training [15,16,18].

Population representation remains a major translational gap. Older adults, adults with severe obesity, patients with sarcopenic obesity, postmenopausal women, men underrepresented in lifestyle programs, racially and ethnically diverse populations, and patients with multiple comorbidities are not consistently represented in nutrition-exercise trials [21,23,24,68,99]. Response to protein-supported concurrent training may differ by age, sex, menopausal status, ethnicity, socioeconomic constraints, baseline fitness, obesity severity, comorbidity burden, pain, medication exposure, and access to food or supervised exercise [99,100,101]. Sex- and gender-related factors require more explicit attention because fat distribution, appetite regulation, cardiometabolic risk, physical activity behavior, medication response, caregiving burden, and barriers to care may differ between groups, while subgroup analyses are often underpowered or exploratory [100,101]. Future studies would be strengthened by prespecified clinically relevant subgroup analyses and by avoiding interpretation of heterogeneous populations as interchangeable when sample size does not support that assumption [99,100,101].

The rise of incretin-based pharmacotherapy creates a particularly urgent research context. GLP-1 receptor agonists and dual incretin therapies can produce substantial reductions in body weight and fat mass, while body-composition analyses indicate that lean mass may also decline during treatment [47,48,84]. The functional meaning of these changes remains uncertain and likely depends on age, baseline muscle reserve, frailty risk, protein intake, resistance training, and the speed and magnitude of weight loss [4,7,8,9]. Current research has not defined the optimal combination of protein dose, resistance-training volume, aerobic dose, and monitoring strategy during GLP-1- or tirzepatide-associated weight loss [38,47,48,84]. Trials are needed to test whether early protein adequacy, progressive resistance training, aerobic conditioning, and functional monitoring preserve strength, mobility, fatigue resistance, and quality of life during pharmacotherapy-induced weight reduction [38,47,48,84]. A related priority exists after bariatric surgery, where rapid weight loss, reduced intake capacity, micronutrient risk, and fat-free mass loss require integrated nutrition and exercise strategies [45,46].

Implementation science represents a central research need in this literature. Diet and exercise adherence are often reported inconsistently, and dropout reasons may not distinguish lack of efficacy from adverse symptoms, pain, stigma, cost, transportation barriers, food insecurity, time constraints, or low perceived competence [60,90,92]. A physiologically coherent intervention has limited clinical value when patients cannot sustain it. Future studies therefore need to report prescribed versus achieved exercise dose, resistance-training progression, protein target achievement, supplement adherence, dietary displacement, gastrointestinal tolerance, injuries, pain flares, and reasons for attrition [90,92]. Cost, feasibility, intervention burden, supervision requirements, and value-for-money analyses are also necessary if protein-supported concurrent training is to be implemented beyond controlled efficacy settings [90,92]. In this sense, feasibility is an outcome rather than a practical afterthought. A program that only works with intensive supervision, expensive supplements, or highly selected participants may have limited public-health relevance.

Precision nutrition and phenotype-based exercise prescription are promising but not ready to replace clinical judgment. Genotype, microbiome, metabolomics, glycemic phenotype, appetite traits, circadian biology, physical activity behavior, and socioeconomic context may eventually refine lifestyle prescription, but current evidence is insufficient to support algorithmic protocols as routine obesity care. A more practical near-term strategy is to stratify patients using visible clinical phenotypes: sarcopenic-obesity risk, low cardiorespiratory fitness, high visceral adiposity, insulin resistance, CKD, incretin-associated appetite suppression, post-bariatric anatomy, pain-limited mobility, and food insecurity [5,6,32,37,38,39]. Adaptive interventions could then adjust protein targets, training modality, supervision, behavioral support, and monitoring intensity according to early response, tolerance, adherence, and functional change [90,92]. The next generation of studies can evaluate higher-protein dietary strategies and concurrent exercise as integrated body-composition and function-preserving interventions, not as isolated weight-loss tools [3,4,21,23,24,68]. Priority designs include multicenter pragmatic randomized trials, longer follow-up with maintenance phases, standardized body-composition protocols, direct functional outcomes, regional and ectopic fat measures, diverse recruitment, and embedded implementation analyses [91,92,93,94,95,96,97,98,99,100,101,102,103,104]. These designs are needed to determine which patients benefit most, which intervention components are essential, which outcomes are meaningful, and how combined nutrition-exercise strategies can be delivered safely, sustainably, and equitably in routine obesity care.

## 9. Limitations of This Narrative Review

This review is limited by its narrative design. The targeted search supported a clinically oriented synthesis but did not include a reproducible systematic search strategy, duplicate screening, formal risk-of-bias assessment, quantitative grading of evidence, or pooled effect estimation. The studies and guidance documents discussed here also differ in population, age, adiposity phenotype, renal status, bariatric history, pharmacotherapy exposure, protein dose, weight denominator, energy deficit, exercise modality, supervision, adherence, and outcome measurement. Evidence from older adults, sarcopenia, sports nutrition, bariatric surgery, chronic kidney disease, and incretin-treated populations informs clinical interpretation, but it is not equivalent to direct trial evidence in all adults with obesity. Body-composition and functional outcomes are also related but noninterchangeable; lean mass, skeletal-muscle mass, muscle quality, strength, mobility, cardiorespiratory fitness, and patient-centered function may change differently during weight loss. These limitations support phenotype-sensitive interpretation rather than a single protein target or exercise prescription across obesity phenotypes.

## 10. Conclusions

Higher-protein nutrition combined with structured exercise is best understood as a strategy for improving the quality of weight loss in adults with obesity, not as a reliable method for maximizing total weight reduction. This should not be interpreted as a population-wide recommendation for unrestricted protein escalation, but rather as a phenotype-sensitive strategy for patients in whom protein adequacy may support lean-tissue preservation, training adaptation, or functional resilience during weight loss. Its clinical value lies in helping shift attention from the scale alone toward fat-mass reduction, preservation of muscle-related function, cardiorespiratory fitness, metabolic health, and physical capacity. The rationale for this approach is coherent but remains proportional to the evidence. Protein adequacy may support satiety, dietary adherence, and attenuation of lean-tissue loss during energy restriction, while resistance training provides the mechanical stimulus required for strength and muscle maintenance. Aerobic and interval-based exercise add cardiorespiratory and metabolic stimuli that complement protein adequacy. These effects are complementary, but they do not establish a single optimal protein dose, training sequence, timing strategy, or combined protocol for all adults with obesity.

The most persuasive clinical argument is functional preservation. Lean mass, skeletal-muscle mass, muscle quality, strength, mobility, and physical performance are related but not interchangeable outcomes; preserving one does not guarantee preservation of the others. For this reason, obesity interventions that combine nutrition and exercise are evaluated using body composition alongside functional measures such as grip strength, chair-rise performance, gait speed, aerobic capacity, fatigue, adverse events, and patient-centered feasibility. Clinical application remains phenotype-sensitive. Older adults, patients with sarcopenic-obesity risk, individuals with low baseline fitness, patients undergoing rapid pharmacological or surgical weight loss, and those with renal disease or limited dietary tolerance may require closer monitoring and more cautious adjustment of protein targets, resistance training, aerobic conditioning, and progression.

The field now needs longer, pragmatic, and better-characterized trials that test protein-supported concurrent training as an integrated body-composition and function-preserving strategy. The remaining questions concern not only whether combined interventions reduce weight, but which patients benefit most, which components are essential, which outcomes matter clinically, and how these strategies can be delivered safely, sustainably, and equitably in routine obesity care.

## Figures and Tables

**Table 1:** Higher-protein nutrition in obesity: clinical uses, limits, and interpretation.

Clinical Domain	What the Evidence Can Support	Interpretive Limits	Key References
Protein target definition	Protein prescriptions specify the dose and the weight reference used, because g/day, g/kg actual body weight, g/kg adjusted or target body weight, percentage of energy, and supplement dose are not interchangeable. Achieved intake is considered separately from prescribed intake.	The term “high-protein diet” does not represent a uniform intervention across trials or clinical settings. Comparable labels may reflect different biological exposures.	[20,21,32]
Practical range during weight loss	Approximately 1.2–1.6 g/kg/day using an appropriate weight reference can be used as a pragmatic working range for many adults with obesity undergoing intentional weight loss.	This range is not a universal requirement for all adults with obesity and is not calculated uncritically from actual body weight in severe obesity.	[20,21,32]
Satiety and dietary adherence	Higher-protein meals may improve fullness and help some patients tolerate energy restriction.	Appetite support is not equivalent to reliable long-term superiority for total weight loss. Short-term satiety does not prove durable adherence or sustained fat loss.	[21,22]
Lean-tissue preservation	Higher protein intake can attenuate muscle-mass loss during weight reduction in adults with overweight or obesity.	Preserved lean mass is not automatically equivalent to preserved strength, muscle quality, mobility, or physical function.	[3,4,5,6,21]
Protein quality and distribution	Total daily intake remains primary; protein quality and distribution may be more relevant in older adults, low-intake states, anabolic resistance, or reduced meal size.	Protein timing is secondary to total intake, training consistency, and overall diet quality. Supplement use does not substitute for assessment of whole-diet quality.	[20,33,38,40,41]
Safety and tolerability	Protein targets account for renal status, post-bariatric gastrointestinal anatomy, incretin-related appetite suppression, gastrointestinal tolerance, diet quality, and clinical goals.	Higher protein is not uniformly safe, necessary, or beneficial for every obesity phenotype. Unsupervised escalation is particularly inappropriate in renal-risk or low-tolerance contexts.	[20,32,37,38,39,45,46]

**Table 2:** Exercise modalities in obesity: targets, practical use, and cautions.

Modality	Main Clinical Role	Practical Use in Obesity Care	Main Cautions	Key References
Resistance training	Supports strength, muscle function, lean-tissue preservation, mobility, and glucose-disposing tissue capacity.	Begin with major movement patterns and large muscle groups on at least two nonconsecutive days per week; progress effort, sets, and complexity as technique and recovery improve.	Do not judge effectiveness only by scale weight or lean-mass change; monitor pain, technique, fall risk, excessive soreness, and confidence. Functional outcomes may improve even when lean-mass change is small.	[15,16,18,20,51,52,53]
Moderate-intensity aerobic training	Improves cardiorespiratory fitness, supports adiposity reduction, and contributes to cardiometabolic health.	Use repeatable low-impact or tolerated modes; progress toward ≥150 min/week when feasible, with higher volumes considered for adiposity reduction or weight-loss maintenance when tolerated.	Avoid abrupt volume increases in patients with pain, dyspnea, neuropathy, severe deconditioning, or very high body mass. Compensatory appetite or activity changes may blunt weight loss despite fitness gains.	[10,11,12,13,18,49,50,56]
Concurrent training	Integrates aerobic and resistance stimuli to address adiposity, fitness, strength, function, and cardiometabolic risk.	Resistance and aerobic exercise may be performed on separate days or in the same session; sequence follows the patient’s priority, fatigue, access, and adherence.	Best interpreted as an integrated option for addressing several outcomes within one program. Time demand and fatigue may reduce feasibility in some patients.	[12,17,18,19,49,55]
HIIT	May provide a time-efficient fitness and cardiometabolic stimulus for selected patients.	Consider only after screening, preparatory conditioning, and assessment of tolerance; low-impact modalities may reduce orthopedic stress.	Not mandatory and not broadly superior; avoid unsupervised vigorous intervals in unstable or high-risk patients. Protocol heterogeneity limits generalization.	[14,18,54,61,62,63]
Mobility, balance, and functional training	Supports gait, chair-rise capacity, movement confidence, fall-risk reduction, and daily-task performance.	Integrate into warm-up, resistance sessions, home practice, or rehabilitation-style progression when mobility is limited.	Complements rather than replaces progressive aerobic and resistance stimuli unless clinical limitations require a preparatory phase.	[5,6,12,13,18,60]

**Table 3:** Selected incretin-based obesity pharmacotherapy trials relevant to body composition and function-preserving strategies.

Study/Context	Intervention	Population	Body-Weight and Body-Composition Findings	Limitations
STEP 1 DXA substudy [82]	Semaglutide 2.4 mg once weekly plus lifestyle intervention.	Adults with overweight or obesity without diabetes.	Reduced body weight, total fat mass, and visceral fat mass; lean body mass also decreased, but the lean-body-mass-to-fat-mass ratio improved.	Exploratory DXA substudy; limited assessment of muscle quality, strength, physical function, protein intake, or training exposure.
STEP 5 [81]	Semaglutide 2.4 mg once weekly for 104 weeks.	Adults with overweight or obesity without diabetes.	Produced sustained weight loss over two years.	Body composition and functional muscle outcomes were not primary endpoints.
SURMOUNT-1 DXA substudy [9]	Tirzepatide once weekly.	Adults with obesity or overweight without diabetes.	Reduced body weight, fat mass, visceral fat mass, and waist circumference; lean mass also declined, while overall body composition improved.	DXA-based analysis; limited information on muscle quality, strength, physical function, protein intake, or resistance training.
SELECT [83]	Semaglutide 2.4 mg once weekly.	Adults with overweight or obesity and established cardiovascular disease without diabetes.	Reduced major adverse cardiovascular events in a high-risk population.	Cardiovascular-outcome trial; not designed to assess body composition, protein adequacy, or exercise-based lean-mass preservation.

This table summarizes selected pharmacotherapy evidence relevant to interpreting lean soft-tissue change during high-magnitude weight loss. It is not a systematic comparison of anti-obesity medications. DXA-derived lean mass is not synonymous with contractile skeletal muscle, muscle quality, strength, or physical function. Abbreviations: DXA, dual-energy X-ray absorptiometry.

**Table 4:** Phenotype-sensitive priorities for protein-supported concurrent training in obesity.

Clinical Context	Practical Priority	Protein Considerations	Exercise Considerations	Key References
Adult with obesity during intentional energy restriction	Improve fat-loss quality while reducing avoidable lean-tissue loss.	Consider approximately 1.2–1.6 g/kg/day using an appropriate weight reference; assess achieved intake, diet quality, and tolerance. Avoid treating the target as meaningful unless actual intake is feasible and sustained.	Combine progressive aerobic activity with resistance training when feasible; monitor waist, function, and fitness, not weight alone.	[3,4,12,13,20,21,32]
Older adult or sarcopenic-obesity risk	Preserve strength, mobility, balance, and independence during weight reduction.	Prioritize adequate total protein, protein quality, and distribution; account for anabolic resistance, appetite, renal status, and frailty risk.	Emphasize progressive resistance training, functional tasks, balance, and tolerable aerobic conditioning. Avoid aggressive weight-loss goals if they compromise intake, recovery, or function.	[4,5,6,33,40,53]
Structured resistance or concurrent training	Support adaptation to training while maintaining diet quality.	Sport-nutrition ranges such as 1.4–2.0 g/kg/day may be informative for exercising individuals, but translation to clinical obesity care remains cautious.	Progress resistance and aerobic stimuli according to symptoms, recovery, confidence, and adherence rather than theoretical sequencing alone.	[17,18,20,21]
GLP-1 receptor agonist or tirzepatide-associated weight loss	Monitor intake, tissue change, and function during high-magnitude weight loss.	Plan protein distribution when early satiety, nausea, reduced intake, constipation, or food aversion limits spontaneous intake. Assess total diet quality, hydration, and micronutrient risk, not protein alone.	Track resistance-training participation, aerobic conditioning, fatigue, strength, and mobility; do not assume pharmacological weight loss preserves function.	[7,9,38,47,48,84,86]
Post-bariatric surgery	Maintain protein intake and functional capacity despite reduced intake capacity.	Commonly cited targets include at least 60–80 g/day or approximately 1.1–1.5 g/kg ideal body weight/day, individualized by procedure, phase, tolerance, and follow-up.	Use gradual resistance and aerobic progression as intake, hydration, symptoms, and surgical recovery allow. Exercise progression follows medical clearance and nutrition tolerance rather than a generic timetable.	[39,45,46,87,88,89]
Chronic kidney disease or high renal-risk profile	Avoid unsupervised protein escalation.	KDIGO advises avoiding high protein intake above 1.3 g/kg/day in adults with CKD at risk of progression; individualize with nephrology or renal-dietetics input when appropriate.	Exercise remains important and is adapted to comorbidity burden, symptoms, anemia, fatigue, and cardiovascular risk.	[37,62,63,90]

The contexts shown are examples of phenotype-sensitive adaptation rather than mutually exclusive categories. Patients may fit more than one context, and protein or exercise targets are adjusted according to safety, tolerance, clinical trajectory, and monitoring data. Abbreviations: CKD, chronic kidney disease; GLP-1, glucagon-like peptide-1; g/kg, grams per kilogram; KDIGO, Kidney Disease: Improving Global Outcomes.

## Data Availability

No new data were created or analyzed in this study. Data sharing is not applicable to this article.
